# Novel Method of Extracting Teeth

**Published:** 1874-09

**Authors:** 


Novel Method of Extracting Deciduous Teeth.—* * * If we desired to extract a deciduous molar for a timid child the rubber ring furnished the most covenient and ready means of doing it without pain, and to the great surprise and gratification of our little patient. All we found to be necessary in the case was to slip one of the rings over the tooth, force it gently under the gum, and dismiss our patient with the injunction not to remove it. The tooth would gradually loosen, and finally fall out, the rubber ring having surely, silently and patiently done the work of the dreaded forceps.—*Dental Journal.*

